# The prognostic and predictive value of the albumin-bilirubin score in advanced pancreatic cancer

**DOI:** 10.1097/MD.0000000000020654

**Published:** 2020-07-10

**Authors:** Tie-Ning Zhang, Ruo-Han Yin, Li-Wei Wang

**Affiliations:** a Nanjing Medical University, Nanjing; b Department of Radiation Oncology, Shanghai General Hospital, Shanghai Jiao Tong University School of Medicine; c Department of Oncology, Renji Hospital, School of Medicine, Shanghai Jiaotong University, Shanghai Cancer Institute, Shanghai, China.

**Keywords:** advanced pancreatic cancer, albumin-bilirubin score, palliative chemotherapy, predictive biomarker, prognostic biomarker

## Abstract

Albumin-bilirubin (ALBI) showed its prognostic and predictive value in hepatobiliary disease like hepatocellular carcinoma. However, little has been known about its role in pancreatic cancer.

In this retrospective study, 149 patients with advanced pancreatic cancer (APC) treated in the Shanghai General Hospital from January 2009 to December 2014 were enrolled as the training cohort and 120 patients treated from January 2015 to December 2018 were taken as the validation cohort. We generated the ALBI score according previous studies. The correlations between ALBI and clinicopathological parameters were evaluated with the Pearson Chi-square test. Kaplan–Meier method and log-rank test were conducted to determine the correlation between ALBI and overall survival (OS). Then we used Cox regression model to investigate the prognostic significance of ALBI. We further assessed retrospectively whether ALBI score could be used to identify combination therapy candidates for APC.

Eastern Cooperative Oncology Group Performance Status, hemoglobin, aspartate aminotransferase, and alanine aminotransferase were found to be significantly correlated with ALBI. Kaplan–Meier analysis showed that the median OS in patients with a pretreatment ALBI ≥−2.6 was 7.0 months, which was significantly shorter than OS of patients with a ALBI <−2.6 (13.0 months, *P* = .001). ALBI was independently correlated with OS in multivariate analysis. In the subgroup analysis, ALBI showed significant prognostic value in patients with liver metastasis but not those without liver metastasis in all 3 cohorts. In addition, only in the group with ALBI <−2.6, patients receiving combination therapy showed better prognosis than those receiving monotherapy.

In conclusion, ALBI was a promising prognostic biomarker in APC with liver metastasis. ALBI also showed predictive value in identifying combination therapy candidates for patients with APC.

## 1 Introduction

Pancreatic cancer is a lethal malignancy that causes approximately 432,242 cancer-related deaths worldwide in 2018.^[1]^ Despite improvements in survival for most cancer types in the last decade, pancreatic cancer is falling behind due to limited progress in diagnostic methods and effective targeted therapeutic interventions.^[2]^ Although various clinical trials showed combination therapy boasted better efficacy than monotherapy in advanced pancreatic cancer (APC), the side effects of combination therapy are usually much severer than monotherapy and many patients cannot tolerate the side effects of combination chemotherapy, such as FOLFIRINOX.^[3,4]^ Identification of defined patient groups with potential biomarkers may help select therapy and improve the prediction of survival.^[5]^ Thus, in the last decade, many studies were conducted to identify diagnostic and prognostic biomarkers in pancreatic cancer but few of them were introduced into clinical practice.^[6,7]^

Jaundice and cachexy are common symptoms in gastrointestinal cancers such as pancreatic cancer.^[8,9]^ Given the levels of bilirubin and albumin can be used to reflect the severity of jaundice, cachexy, and hepatic function, Johnson et al first developed the albumin-bilirubin (ALBI) score to assess liver function in 2015.^[10]^ Subsequently, a series of studies investigated the predictive and prognostic values of ALBI in hepatocelluar carcinoma and other hepatobiliary disease such as primary biliary cirrhosis.^[11–15]^ The lower level of ALBI was found to be correlated with better survival and it could be used to identify candidate hepatocellular carcinoma patients for starting regorafenib treatment.^[16]^ In addition, various studies indicate the prognostic value of ALBI in other types of cancer such as gastric cancer.^[17]^ Although Takuki et al found preoperative ALBI grade was a useful prognostic indicator in resectable pancreatic cancer, little has been known about its role in APC.^[18]^

We conducted a multicentral study to evaluate whether the baseline ALBI score could be a potential prognostic and predictive biomarker for APC patients receiving firstline chemotherapy.

## 2 Patients and methods

### 2.1 Patients

From January 2009 to December 2018, 269 patients with locally advanced or metastatic pancreatic cancer (ICD, Tenth Revision, codes C25) enrolled at the Shanghai General Hospital were enrolled. Among them, 149 patients treated from January 2009 to December 2014 were taken as the training cohort and 120 ones treated from January 2015 to December 2018 were taken as the validation cohort. The following inclusion criteria were applied:

(1) with pathologically confirmed pancreatic adenocarcinoma;
(2) without any concurrent cancer at another organ site;
(3) with at least 2 cycles of palliative first-line chemotherapy after first diagnosis;
(4) with complete records of clinicopathological features.

Baseline clinicopathological characteristics of these patients were summarized in Table 1. The laboratory test was performed within 1 to 3 days before chemotherapy. Palliative chemotherapy regimens included monotherapy like gemcitabine or S-1, and combination therapy like gemcitabine plus S-1, gemcitabine plus nab-paclitaxel and FOLFIRINOX.^[19–23]^ Informed consent was obtained from all subjects and this study was approved by the Ethics Committees of Shanghai General Hospital. The methods were carried out in accordance with the relevant guidelines and regulations.

**Table 1:**
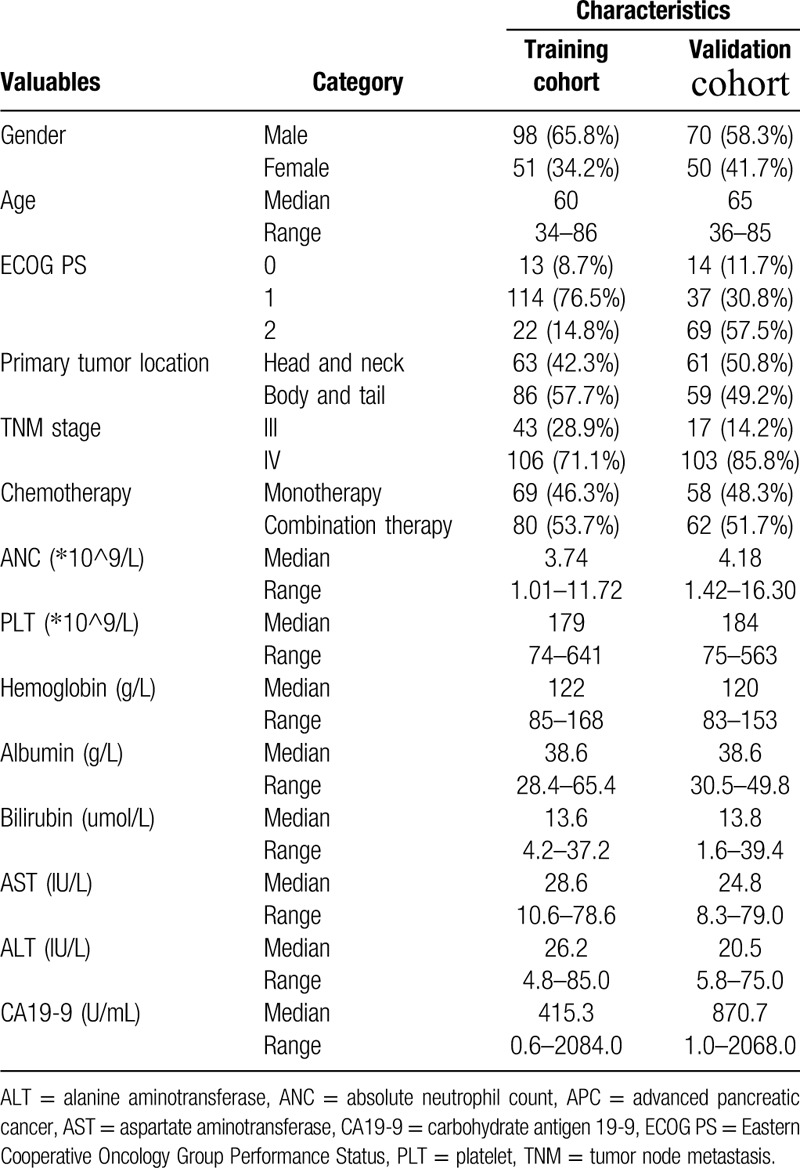
Baseline clinicopathological characteristics of patients with APC.

### 2.2 Cutoff value for ALBI

**Figure 1:**
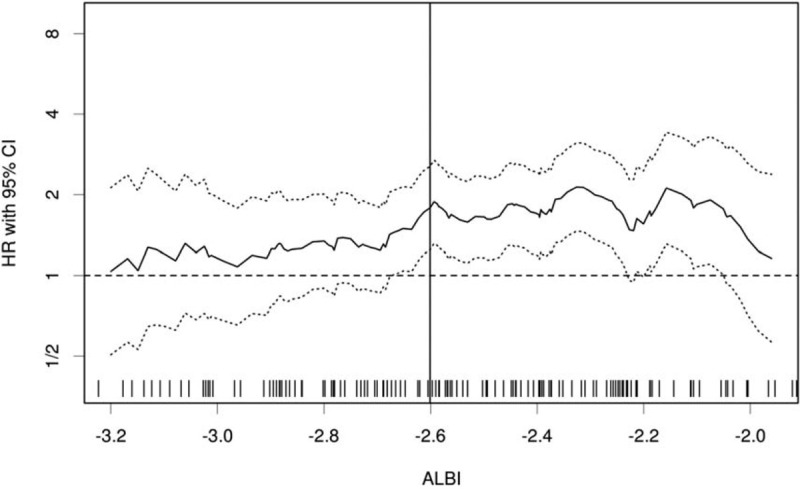
The hazard ratio (HR) including 95% CI for OS according to the cutoff value of the ALBI score in patients with APC. The distribution of the ALBI score is shown as rug plot at the bottom of the figure. The optimal cutoff is marked by a vertical line. ALBI = albumin-bilirubin, APC = advanced pancreatic cancer, CI = confidence interval, OS = overall survival.

ALBI score was calculated as described before: ALBI score = (log10 bilirubin × 0.66) + (albumin × −0.085). With the online biostatistical tool Cutoff Finder, the optimal cutoff value of −2.6 was identified (Fig. 1).^[24]^ The ALBI of −2.6 corresponded to the maximum sum of sensitivity and specificity, which was equivalent to the maximization of Youden J statistics (J = sensitivity + specificity − 1). The optimal cutoff values of absolute neutrophil count (3.965), platelet (280), and hemoglobin (124.5) were also identified with the same method.

### 2.3 Statistical analysis

All statistical analyses were performed with SPSS statistical software (version 21.0, SPSS Inc., Chicago, IL) and State software (version 12.0, StataCorp, College Station, TX). Descriptive statistics were presented as median level and 95% confidence interval (95% CI). For the assessment of correlations between ALBI and other valuables, patients were classified into 2 groups according to gender (male and female), age (≥60 or <60 years), Eastern Cooperative Oncology Group Performance Status (ECOG PS) (0, 1 or 2), primary tumor location (head and neck or body and tail), Tumor node metastasis (TNM) stage (III or IV), chemotherapy (monotherapy or combination therapy), absolute neutrophil count (≥3.965 or <3.965), platelet (≥280 or <280), hemoglobin (≥124.5 or <124.5), carbohydrate antigen 19-9 (CA19-9) (≥1000 or <1000), aspartate aminotransferase (AST) (≥40 or <40), alanine aminotransferase (ALT) (≥40 or <40), and ALBI (≥−2.6 or <−2.6).^[25]^ Comparison between these groups was conducted by using the Pearson Chi-square test. OS was calculated from the date of chemotherapy initiation and terminated on the date of death for any reason or censored on the last follow-up visit. Furthermore, survival analysis was performed with the Kaplan–Meier method and the log-rank test. Cox regression analysis was used to investigate independent prognostic factors for OS. For each factor, we calculated the hazard ratios (HRs) and corresponding 95% CIs. Two-sided *P* < .05 was considered statistically significant.

## 3 Results

### 3.1 Patient characteristics

Baseline clinicopathological characteristics of patients in the training cohort, validation cohort, and testing cohort were summarized in Table 1. In the training cohort, the median age of patients was 60 years (range 34–86). Among them, 98 (65.8%) were male, 127 (85.2%) had relatively good performance status (ECOG PS 0-1), 63 (42.3%) had tumors occurred in the head and neck of the pancreas and 106 (71.1%) had metastatic disease. In addition, 69 (46.3%) and 80 (53.7%) patients were treated with monotherapy and combination therapy, respectively.

### 3.2 Correlation between ALBI and clinicopathological variables

We investigated the correlation between the ratio of lymphocyte to monocyte and other clinicopathological variables in the training cohort (Table 2). ECOG PS (*P* = .009), hemoglobin (*P* < .001), AST (*P* = .002), and ALT (*P* = .011) were found to be significantly correlated with ALBI. However, other clinicopathological characteristics were comparable between 2 groups (*P* > .05 for all).

**Table 2:**
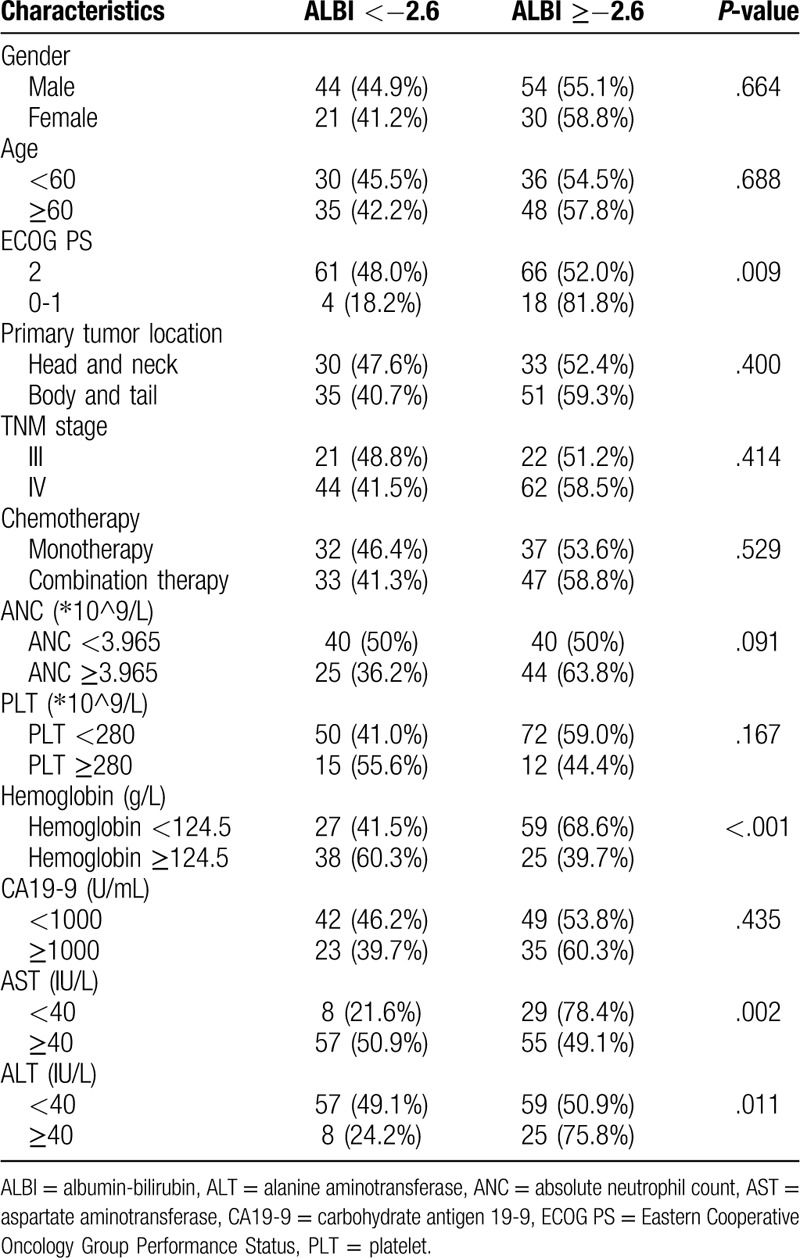
Correlations between ALBI and clinicopathological variables.

### 3.3 Univariate and multivariate analysis of prognostic factors

**Figure 2:**
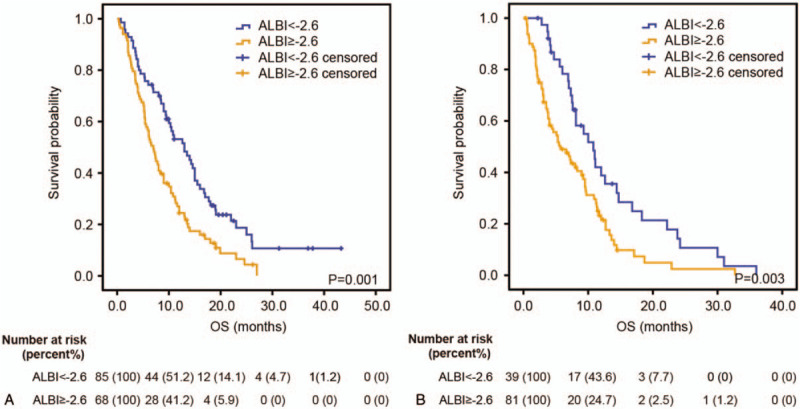
Kaplan–Meier estimates OS according to the ALBI score in both training cohort (A) and validation cohort (B). In the training cohort and validation cohort, the median OS in patients with a pretreatment ALBI >−2.6 was significantly shorter than those of patients with a ALBI <−2.6. ALBI = albumin-bilirubin, OS = overall survival.

Figure 2A demonstrated that in the training cohort, the median OS in patients with a pretreatment ALBI ≥−2.6 was 7.0 (95% CI 5.4–8.6) months, which was significantly shorter than those of patients with a ALBI <−2.6 (13.0 months, 95% CI 9.4–16.6, *P* = .001). In univariate analysis, 5 factors, including ECOG PS (*P* = .003), TNM stage (*P* = .002), CA19-9 (*P* = .011), AST (*P* = .022), and ALBI (*P* = .002), were found to be correlated with OS (Table 3). These factors were subsequently analyzed in multivariate analysis and only TNM stage, CA19-9, and ALBI showed independent prognostic value.

**Table 3:**
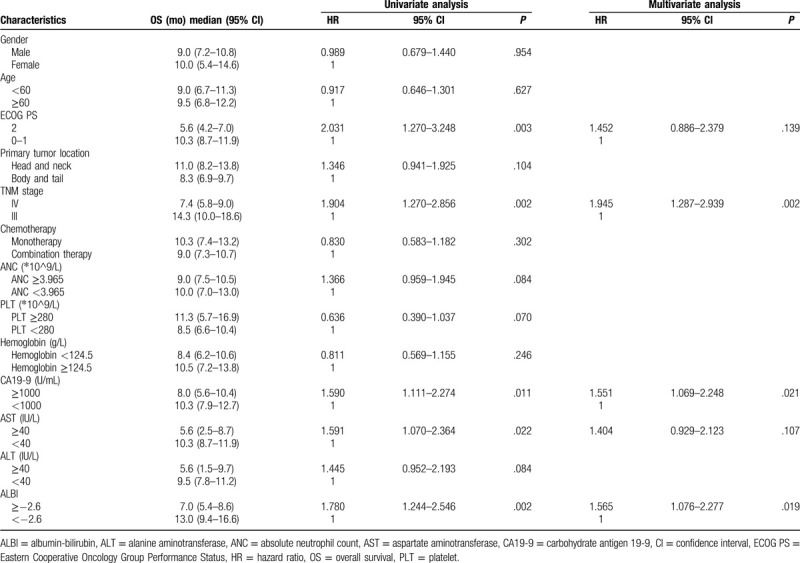
Univariate and multivariate analysis regarding OS in the training cohort.

### 3.4 Predictive value of ALBI in therapeutic decision-making

**Figure 3:**
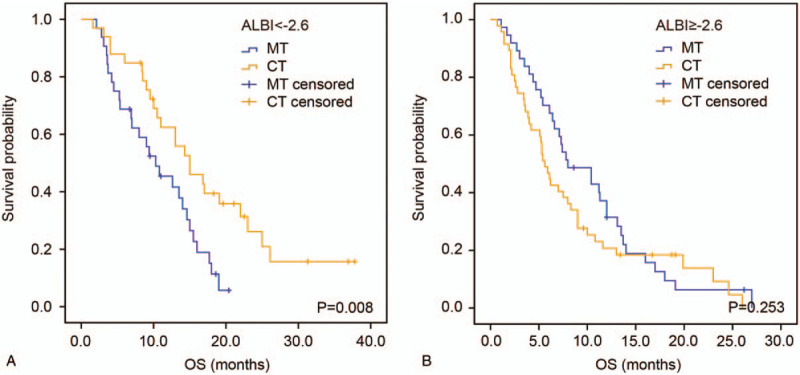
Kaplan–Meier analysis according to the ALBI-based groupings in the training cohort. Kaplan–Meier estimates OS according to chemotherapy regimens in the group of ALBI <−2.6 (A) and group of ALBI >−2.6 (B). ALBI = albumin-bilirubin, CT, combination therapy, MT, monotherapy, OS = overall survival.

To investigate the predictive value of ALBI score in therapeutic decision-making, we divided patients into 2 groups according to ALBI in the training cohort. Intriguingly, in the group with ALBI <−2.6, patients receiving combination therapy had better prognosis than those receiving monotherapy (median OS 15.0 vs 10.3 months, HR: 0.461, 95% CI: 0.255–0.832, *P* = .008, Fig. 3A). In the patients with ALBI >−2.6, there was no significant difference in OS between patients receiving monotherapy or combination therapy (HR: 1.304, 95% CI: 0.824–2.063, *P* = .253, Fig. 3B).

### 3.5 Validation of ALBI score's prognostic and predictive value

**Figure 4:**
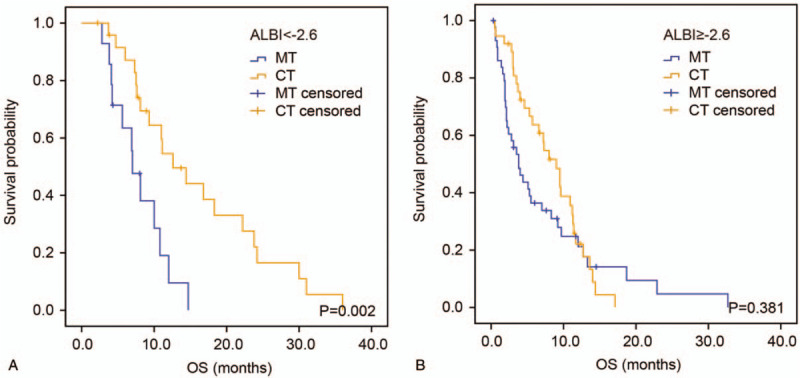
Kaplan–Meier analysis according to the ALBI-based groupings in the validation cohort. Kaplan–Meier estimates OS according to chemotherapy regimens in the group of ALBI <−2.6 (A) and group of ALBI >−2.6 (B). ALBI = albumin-bilirubin, CT, combination therapy, MT, monotherapy, OS = overall survival.

In the validation cohort, the median OS in patients with ALBI ≥−2.6 was 5.7 (95% CI 3.0–8.4) months, which was also shorter than those of patients with ALBI <−2.6 (10.8 months, 95% CI 7.7–13.9, *P* = .003, Fig. 2B). Univariate and multivariate analysis demonstrated that ECOG PS (*P* = .004), TNM stage (*P* = .040), CA19-9 (*P* = .002), and ALBI (*P* = .049), rather than AST, were independently correlated with OS (Table 4). Notably, patients receiving combination therapy also showed better OS than those receiving monotherapy in the group with ALBI <−2.6 (median OS 12.6 vs 7.0 months, HR: 0.296, 95% CI: 0.130–0.675, *P* = .002, Fig. 4A). Likewise, no significant difference was seen in OS between patients receiving monotherapy and combination therapy in the group with ALBI >−2.6 (HR: 0.804, 95% CI: 0.492–1.314, *P* = .381, Fig. 4B).

**Table 4:**
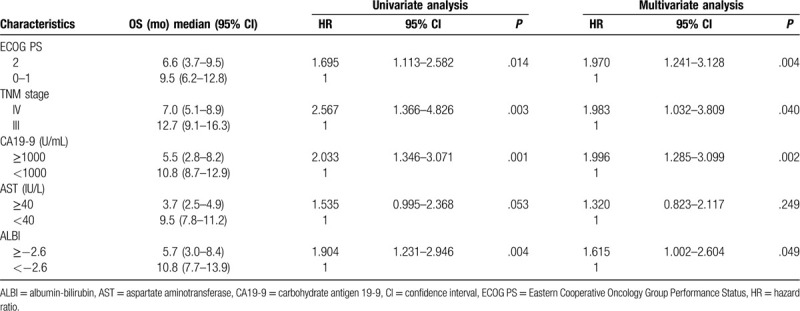
Univariate and multivariate analysis regarding OS in the validation cohort.

### 3.6 Subgroup analysis considering liver metastasis

**Figure 5:**
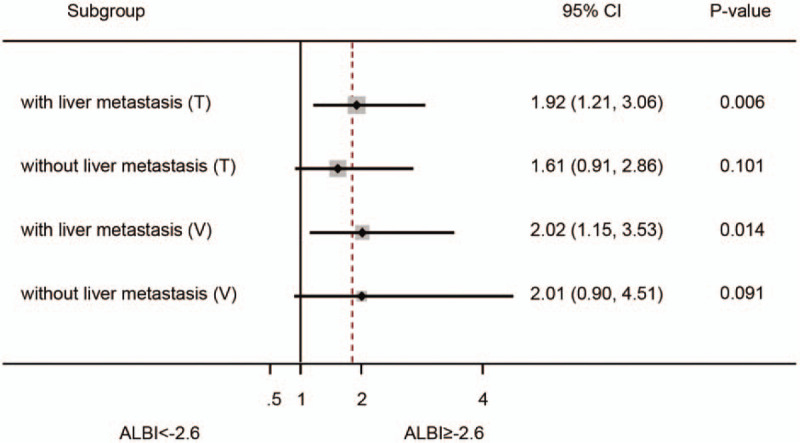
Subgroup analysis to investigate the prognostic value of ALBI score in different status of liver metastasis. ALBI showed significant prognostic value in patients with liver metastasis but not those without liver metastasis in the training cohort and validation cohort. ALBI = albumin-bilirubin, T = training cohort, V = validation cohort.

Given the fact that liver metastasis can affect hepatic reserve function, which is reflected by ALBI, we further investigate ALBI's prognostic value in subgroups of different status of liver metastasis (Fig. 5). In the training cohort, ALBI showed significant prognostic value in patients with liver metastasis but not those without liver metastasis (*P*-value: .006 vs .101). Likewise, similar result was found in the validation cohort (*P*-value: .014 vs .091) and testing cohort (*P*-value: <.001 vs .223).

## 4 Discussion

Up to date, chemotherapy is still the major choice for APC, although there are emerging progress in targeted therapy and immunotherapy worldwide.^[26,27]^ However, the lack of effective biomarkers makes it difficult for clinicians to make precise therapeutic decisions from a variety of chemotherapy regimens.^[7]^ For example, combination therapy showed superiority in prolonging OS or progression free survival than monotherapy, but their severe side effects might be less tolerable for patients. In China, many patients are prone to receive safer treatment with lower toxicity and only few of them can tolerate the side effects of FOLFIRINOX, one of the most effective regimens in the treatment of APC.^[28]^ Thus, there is a need in identifying diagnostic and prognostic biomarkers for pancreatic cancer patients. In the present study, we found ALBI score, which reflected patients’ liver function and nutritional status, was independently correlated with OS in patients with liver metastasis. Furthermore, in both training cohort and validation cohort, ALBI score was a potential predictive biomarker in identifying combination treatment candidates in patients with APC.

Previously, the cutoff value of ALBI was defined as follows: ≤−2.60 (grade 1), >−2.60 to ≤−1.39 (grade 2), and >−1.39 (grade 3). Such value was acquired by calculating the patient-level linear prediction reported by Johnson et al in hepatocellular cancer.^[10]^ However, there was little convincing evidence that it could also be properly applied in other types of disease. In pancreatic cancer, Takuki et al used the same cutoff value of ALBI but they thought it was still necessary to calculate an optimal cutoff value for pancreatic cancer.^[18]^ Intriguingly, we found that the ALBI of −2.6 was also the optimal cutoff value for pancreatic cancer with the online biostatistical tool Cutoff Finder, and such value could apparently distinguish patients with good prognosis from those with poor prognosis. However, more studies are still needed to verify whether −2.6 can be the optimal cutoff value of ALBI regardless of the type of cancer.

In the present study, ECOG PS, hemoglobin, AST, and ALT were found to be significantly correlated with ALBI score. The ALBI score was composed of 2 parts including albumin and bilirubin. Albumin is synthesized in the liver and it can be used reflect people's nutritional status. When patient's liver function is impaired or nutritional status is poor, the serum concentration of albumin will decrease and ALBI score will increase. Meanwhile, the impairment of liver can also cause the secretion and dysregulation of bilirubin, which will lead to an increasing level of circulating bilirubin. Notably, hemoglobin, AST, and ALT can also reflect liver function in some aspects, thus it is rational that the mentioned factors have a significant correlation with ALBI.

Kaplan–Meier analysis demonstrated that the median OS in patients with a pretreatment ALBI ≥−2.6 was significantly shorter than those of patients with a ALBI <−2.6 (*P* = .001). In addition, multivariate analysis showed ALBI was an independent prognostic factor for APC. Given the fact that liver metastasis may largely affect hepatic reserve function, which is reflected by the level of ALBI, we further conducted subgroup analysis. Intriguingly, ALBI showed significant prognostic value only in group of patients with liver metastasis in the 2 cohorts. Because liver metastasis will inevitably impair hepatic function and the straightforward utility of ALBI score is to assess hepatic reserve function, such results suggest that ALBI may be more suitable to be applied in cancer patients with liver metastasis.

We also investigated the predictive value of ALBI for patients with APC. We found that in the group of patients with ALBI <−2.6, those receiving combination therapy had better prognosis than those receiving monotherapy. In contrast, in the patients with ALBI ≥−2.6, there was no significant difference in OS between patients receiving monotherapy or combination therapy. As mentioned before, patients with ALBI <−2.6 meant they were with a lower level of bilirubin and a higher level of albumin, which suggested they had better performance status and liver function. Thus, this kind of patients can tolerate more intensive treatment strategy even with more severe side effects and benefit more from combination therapy.

There are some limitations to be addressed in this study. One of these limitations is that it is a retrospective study, thus the potential of bias exists. Another limitation is that heterogeneous treatments in this study may affect survival although we found there was no significant difference in OS between patients receiving monotherapy or combination therapy. In addition, the baseline bilirubin may be influenced by other confounding factors like chronic cholecystitis. Therefore, multicenter studies with large sample size are warranted to confirm the results.

## 5 Conclusion

ALBI is a promising prognostic and predictive biomarker and can be used to identify combination therapy candidates for patients with APC.

## Author contributions

**Data curation:** Tie-Ning Zhang.

**Formal analysis:** Tie-Ning Zhang, Ruo-Han Yin.

**Methodology:** Tie-Ning Zhang, Ruo-Han Yin.

**Resources:** Tiening Zhang, Li-Wei Wang.

**Supervision:** Li-Wei Wang.

**Validation:** Li-Wei Wang.

**Writing – original draft:** Tie-Ning Zhang, Ruo-Han Yin.

**Writing – review & editing:** Li-Wei Wang.
